# Enterovirus 71 drives TLR2–NF-κB–dependent inflammatory skeletal muscle injury in neonatal BALB/c mice

**DOI:** 10.1128/spectrum.00383-26

**Published:** 2026-09-15

**Authors:** Xinhong Zou, YaXin Li, Xiaomeng Zhang, Honglin Niu, Hairui Zhou, Lei Liu, Guoli Cui, Baixin Wang, Rui Wu

**Affiliations:** 1 Clinical Medicine Department, Jiamusi University 117916, Jiamusi, Heilongjiang Province, China; 2 Basic Medical College of Jiamusi University, Jiamusi University 117916, Jiamusi, Heilongjiang Province, China; Engelhardt Institute of Molecular Biology, Moscow, Russia

**Keywords:** EV-A71, TLR2, NF-κB, inflammation, skeletal muscle, suckling mouse

## Abstract

Enterovirus 71 (EV-A71) can cause inflammatory injury beyond the central nervous system, yet the host pathways driving EV-A71-associated skeletal muscle pathology remain poorly defined. Toll-like receptor 2 (TLR2) is a key innate immune sensor capable of amplifying inflammatory signaling during infection. Here, we established an EV-A71 infection model in 1-day-old BALB/c suckling mice by intraperitoneal inoculation for 3 consecutive days and administered the TLR2 inhibitor C29 intraperitoneally at 6 h post-inoculation. EV-A71 infection induced prominent skeletal muscle pathology characterized by inflammatory cell infiltration, muscle bundle disruption, and dissolution, and reduced myofiber cross-sectional area. Consistently, EV-A71 increased the levels of TLR2, nuclear factor-κB (NF-κB) signaling markers (p65 and phosphorylated p65), the pro-inflammatory cytokine IL-6, and the viral protein VP1 in skeletal muscle. EV-A71 infection was also associated with elevated α-SMA and collagen III, suggesting tissue remodeling in injured muscle. Notably, pharmacological inhibition of TLR2 with C29 markedly alleviated histopathological damage and reduced IL-6 expression in skeletal muscle. Collectively, these findings support a model in which EV-A71 activates a TLR2–NF-κB–linked inflammatory program that contributes to skeletal muscle immunopathology and highlights TLR2 as a tractable host-directed target for mitigating virus-associated skeletal muscle injury.

**IMPORTANCE:**

Virus-associated skeletal muscle injury can cause functional impairment and may worsen outcomes, but actionable host determinants remain limited. Using a neonatal mouse model, we show that enterovirus 71 (EV-A71) infection is accompanied by activation of a Toll-like receptor 2 (TLR2)-linked inflammatory program in skeletal muscle and that pharmacological TLR2 inhibition reduces IL-6 expression and alleviates muscle pathology. These results support the concept that excessive innate immune signaling contributes to muscle immunopathology and identify TLR2 as a potential host-directed intervention point. This work provides a mechanistic framework for understanding and targeting skeletal muscle injury associated with EV-A71 and potentially other viral infections.

## INTRODUCTION

Skeletal muscle accounts for approximately 40% of total body weight in humans and is essential for maintaining homeostasis, enabling movement, and promoting blood circulation (1). Skeletal muscle injury can reduce quality of life and exacerbate disease progression. The etiologies and severity of skeletal muscle damage are diverse, including indirect injuries (e.g., strains), direct injuries (e.g., contusions or lacerations), degenerative disorders (e.g., muscular dystrophy), and infectious myositis caused by viral infection (2). Clinically, infections with various viruses (e.g., influenza virus and coxsackievirus) often lead to pronounced myalgia and fatigue. Although different viruses exhibit distinct tissue tropisms and primarily affect different organs, the innate immune activation and inflammatory cascades they induce frequently converge on several shared key molecular pathways or nodes. Elucidating how viruses trigger inflammatory signaling cascades within skeletal muscle not only deepens our understanding of the pathogenesis of viral myositis but also provides a theoretical basis for mitigating muscle injury associated with a broad spectrum of viral infections.

Enterovirus 71 (EV-A71) is a non-enveloped, positive-sense single-stranded RNA virus belonging to the family Picornaviridae and the genus Enterovirus. Its genome is approximately 7 kb in length and encodes four structural proteins (VP1–VP4) and seven non-structural proteins (2A–2C and 3A–3D) (3, 4). EV-A71 is environmentally stable and highly transmissible, causing diseases such as hand-foot-and-mouth disease (HFMD) and herpangina, with children under 5 years of age being particularly susceptible (5). Clinically, EV-A71 can lead to severe neurological complications; however, in adult patients, its effects on skeletal muscle are often limited to subtle, subclinical manifestations. In contrast, experimental animal models exhibit pronounced myotropism, with EV-A71 infection inducing dramatic limb paralysis and myofiber necrosis. This phenotypic amplification makes the model an ideal platform for dissecting virus-induced inflammatory responses, enabling more precise observation of microscopic molecular lesions that may be obscured in clinical settings.

Current studies suggest that viruses may cause myocyte injury and necrosis either by directly infecting muscle cells or by inducing inflammatory responses. Inflammation underlies a wide range of physiological and pathological processes; it is triggered by diverse noxious stimuli and is generally regarded as an intrinsic protective mechanism that supports tissue repair, maintains internal homeostasis, and preserves organ function. Following viral infection, the host initiates immune responses and releases multiple pro-inflammatory cytokines to eliminate the virus and counteract infection (6). After skeletal muscle injury, inflammatory responses are rapidly activated, involving the dynamic participation of numerous cells and molecular mediators. These responses can facilitate muscle repair but may also aggravate tissue damage (7, 8). Toll-like receptors (TLRs) are pattern-recognition receptors expressed on innate immune cells; upon activation, they drive the production of abundant pro-inflammatory factors, amplify inflammatory cascades, and participate in inflammation and anti-infective immune processes. Among them, Toll-like receptor 2 (TLR2) is a member of the TLR family and mediates host immune and inflammatory responses. TLR2 can regulate the adaptor myeloid differentiation primary response 88 (MyD88), leading to aberrant activation of the downstream transcription factor nuclear factor-κB (NF-κB) and promoting the expression of a series of inflammatory mediators, including IL-6 and IL-8 (9). Notably, IL-6 is an important determinant of muscle function; during acute influenza A virus-induced pneumonia, IL-6 can be released from injured lung tissue and subsequently drive skeletal muscle functional decline (10). When skeletal muscle is damaged, fibroblasts secrete type I, III, and VI collagen, and large numbers of muscle satellite cells are activated. Under chronic inflammation, sustained infiltration of macrophages and neutrophils, together with aberrantly elevated cytokines such as TGF-β and IL-6, can induce satellite cells to transdifferentiate into myofibroblasts, which secrete excessive extracellular matrix (11, 12).

In this context, chronic inflammation not only drives tissue injury and functional deterioration but may also promote fibrogenesis, thereby exacerbating structural disruption of the affected tissue. Therefore, an in-depth investigation of the role of TLR2 in inflammation and infection—particularly the mechanisms by which it mediates EV-A71-induced muscle injury—may facilitate the development of novel therapeutic strategies to attenuate inflammatory responses and tissue damage, ultimately improving patient survival and quality of life. To this end, we employed EV-A71-infected BALB/c neonatal mice as the experimental model and established a skeletal muscle injury model in which EV-A71 infection was blocked by TLR2 inhibition. This approach aimed to elucidate the novel mechanisms by which the TLR2/NF-κB signaling pathway contributes to the initiation and progression of EV-A71-induced skeletal muscle injury, thereby providing critical experimental support for identifying shared signal transduction pathways and potential therapeutic targets underlying broad-spectrum virus-associated muscle injury.

## MATERIALS AND METHODS

### Animals

All experimental BALB/c mice were purchased from Changchun Yisi Laboratory Animal Technology Co., Ltd. [license no. SCXK (Ji)−2020–0002, Changchun, China]. Specific pathogen-free (SPF) BALB/c mice (body weight 18 g–22 g) were used, including 20 males and 40 females. Animals were housed in a temperature-controlled room within the SPF barrier facility of the university animal center under a 12 h light/dark cycle, at 21°C–25°C with 55 ± 5% relative humidity, with *ad libitum* access to food and water. Mice were acclimatized for 1 week prior to experiments, after which females and males were randomly paired at a 2:1 ratio. One-day-old BALB/c pups were randomly assigned to three groups: a control group (*n* = 36), an EV-A71 model group (*n* = 36), and a TLR2 inhibitor (C29) group (*n* = 15). The control and model groups were further randomly subdivided into four time-point subgroups (5, 7, 10, and 14 days), with nine pups per subgroup. One-day-old BALB/c pups were randomly divided into three groups: the control group (*n* = 36), the EV-A71 model group (*n* = 36), and the TLR2 inhibitor (C29) group (*n* = 15). The control and model groups were further randomly divided into four time-point subgroups (days 5, 7, 10, and 14), with nine pups in each subgroup. Starting from day 1 post-natal, newborn BALB/c mice were administered intraperitoneal injections of an EV-A71 viral suspension for 3 consecutive days. The viral titer was 10⁴^.^¹² TCID_50_/mL, with 25 μL administered per gram of body weight, equivalent to 25 × 10³ μL/kg of body weight. Given that the body weight of newborn mice is approximately 1 g–3 g, the volume injected per mouse per dose was approximately 25 μL–75 μL, equivalent to approximately 330–990 TCID_50_ of EV-A71 virus per mouse per dose, thereby establishing an EV-A71 infection model in newborn mice. In the C29 group, pups were administered C29 (20 mg/kg, intraperitoneally) 5 h prior to each viral inoculation, for 10 consecutive days. On day 10, the neonatal mice were euthanized under CO₂ anesthesia, and skeletal muscle tissues were rapidly harvested immediately following anesthesia (13).

### Cell

Human rhabdomyosarcoma (RD) cells were kindly provided by the Chinese Center for Disease Control and Prevention and were cultured in modified Dulbecco’s Eagle’s Medium (Gibco, Grand Island, New York, USA), supplemented with 10% inactivated fetal bovine serum (FBS; Gibco), 100 U/mL penicillin, and 100 μg/mL streptomycin (Gibco), and cultured at 37°C in a humidified environment containing 5% CO₂.

### Virus

The EV-A71 strain used in this study was EU703812, originally isolated in 2008 from an infected patient in Fuyang, China, and provided by the Chinese Center for Disease Control and Prevention.

### Virus concentration

Virus inoculation was carried out when the RD cell density reached approximately 80%–90%. The EV-A71 virus stock solution was diluted to the required concentration using cell maintenance medium containing 2% FBS, and inoculated into RD cells at a rate of 4 mL–5 mL of virus dilution per 25 cm² culture flask. When the cytopathic effect in the RD cells exceeded 80%, the infected cell suspension was collected. After repeated freeze-thaw cycles, the suspension was centrifuged at 4°C and 3,000 rpm for 15 min to remove cell debris, and the supernatant was collected. The supernatant was then transferred to a 30 kDa ultrafiltration tube and centrifuged at 4°C and 3,800 × *g* for approximately 30 min. The viral concentrate was collected, filtered through a 0.22 μm sterile filter membrane, aliquoted, and stored at −80°C for future use (14).

### Main reagents and instruments

#### Main reagents and antibodies

C29 (HY-100461; MedChemExpress, USA), Triton X-100 (926488A; Invitrogen, USA), and DAPI staining solution (14G17B77; Boster Biological Technology, China) were used in this study. The primary antibodies included an antibody against phosphorylated NF-κB p65 (p-p65; sc-135769; Santa Cruz Biotechnology, USA), an antibody against NF-κB p65 (sc-515045; Santa Cruz Biotechnology, USA), an anti-collagen III antibody (HA720050; HUABIO, China), an anti-EV-A71 VP1 antibody (MAB1255-M08; Abnova, China), an anti-IL-6 antibody (DF6087; Affinity Biosciences, USA), and an anti-GAPDH antibody (GB15004; Servicebio, China). The secondary antibodies included HRP-conjugated goat anti-mouse IgG (H+L) (BA1050; Boster Biological Technology, China) and HRP-conjugated goat anti-rabbit IgG (H+L) (BA1054; Boster Biological Technology, China).

#### Main instruments

The main instruments used in this study included a high-speed refrigerated centrifuge (Allegra X-64R, Beckman Coulter, USA), a laser microdissection system (LMD6000, Leica Microsystems, Germany), a laser scanning confocal microscope (FV1000, Olympus, Japan), a pathological image acquisition and analysis system (DM4000B, Leica Microsystems, Germany), a fluorescence microscope (DM1400B, Leica Microsystems, Germany), and an automatic chemiluminescence/fluorescence imaging analysis system (Tanon 4200, Tanon Science & Technology Co., Ltd., Shanghai, China).

#### Image acquisition and analysis

Chemiluminescence/fluorescence signals were detected using an automatic chemiluminescence/fluorescence imaging analysis system (Tanon 4200, Tanon Science & Technology Co., Ltd., Shanghai, China). The intensity of each target protein band was quantified by analyzing grayscale values using ImageJ software (version 1.54f; National Institutes of Health, Bethesda, MD, USA).

### Body mass and disease score detection in suckling mice

After successful modeling, the weight of BALB/c mice was observed and recorded every day, the clinical symptom scores were evaluated, and the mortality rate was recorded. Clinical scores were defined as follows: 0, no signs of disease; 1, unilateral or bilateral hindlimb weakness and/or unsteady gait; 2, unilateral hindlimb paralysis; 3, bilateral hindlimb paralysis; 4, paralysis of both forelimbs and hindlimbs; 5, death (15).

### Skeletal muscle hematoxylin-eosin (HE) staining, picrosirius red staining, and Masson staining

The skeletal muscle was dehydrated with ethanol step by step, embedded in paraffin, and sliced. It was stained with HE, picrosirius red, and Masson stain, and the slices were sealed. The pathological changes of skeletal muscle tissue were observed under an optical microscope.

### Western blot analysis of skeletal muscle-related proteins

Skeletal muscle tissue was homogenized in lysis buffer using a tissue homogenizer, and the supernatant was collected after centrifugation for protein extraction. After SDS-PAGE, proteins were transferred to PVDF membranes and blocked with 5% skim milk powder for 1 h 30 min. TLR2 (1:2,000), NF-κB (P65) (1:2,000), NF-κB (P-P65) (1:2,000), IL-6 (1:3,000), collagen III (1:1,000), α-SMA (1:2,000), EV-A71 VP1 (1:2,000), and GAPDH (1:1,000) antibodies were incubated at 4°C for 12 h, the secondary antibody was incubated at 37°C for 1 h, and the exposure solution was incubated in the dark. The gray value was calculated by the gel imaging system (Tanon Company, America), and the relative protein expression in each group was analyzed by taking pictures.

### Immunofluorescence

Paraffin sections were deparaffinized and subjected to heat-induced antigen retrieval in 10 mM citrate buffer (pH 6.0) using a microwave oven at medium-low power for 20 min, followed by natural cooling to room temperature. After blocking with 5% BSA, the sections were incubated with primary antibodies against TLR2 (1:100), NF-κB (P65) (1:100), NF-κB (P-P65) (1:100), IL-6 (1:200), α-SMA (1:100), collagen III (1:200), and EV-A71 VP1 (1:100) overnight at 4°C. After washing with PBS, Cy3-conjugated sheep anti-rabbit IgG and Alexa Fluor 488-conjugated sheep anti-mouse IgG secondary antibodies were added and incubated at 37°C in the dark for 1 h. Subsequently, DAPI staining solution was applied, and fluorescence images were captured using a fluorescence microscope after mounting. The results were analyzed using ImageJ software.

### RNA extraction and qRT-PCR analysis

Total RNA was extracted from skeletal muscle tissue using Trizol reagent. cDNA was synthesized by reverse transcription using a cDNA kit (Takara). qRT-PCR was performed on a LightCycler 96 real-time PCR system using the TB Green Premix Ex PCR kit (Takara). The primer sequences used for RT-qPCR analysis are listed in Table 1.

**TABLE 1:** Primer sequences used in this study^*a*^

Target gene	Species	Accession no.	Forward primer sequence (5′–3′)	Reverse primer sequence (5′–3′)
*VP1*	*EV-A71*	EU703812	AGTATGATTGAGACTCGGTG	GCGACAAAAGTGAACTCTGC
*Tlr2*	*Mus musculus*	NM_011905.3	ACAGCAAGGTCTTTCCTGGTTCC	GCTCCCTTACAGGCTGAGTTCT
*Il6*	*Mus musculus*	NM_001314054.1	CAACGATGATGCACTTGCAGA	GTGACTCCAGCTTATCTCTTGGT
*Rela* *(NF-κB p65)*	*Mus musculus*	NM_009045.5	CTTCCTCAGCCATGGTACCTCT	CAAGTCTTCATCAGCATCAAACTG
*Gapdh*	*Mus musculus*	NM_008084.4	CATCACTGCCACCCAGAAGACTG	ATGCCAGTGAGCTTCCCGTTCAG

^*a*^ The EV-A71 strain used in this study was kindly provided by the Center for Disease Control and Prevention and maintained in our laboratory. The GenBank accession number of the EV-A71 genome was EU703812. The VP1 primers were designed based on this EV-A71 genome sequence, and the mouse gene primers were designed according to the corresponding Mus musculus RefSeq mRNA sequences in the NCBI database.

#### Statistical analysis

Experimental data are expressed as the mean ± standard error of at least three independent experiments. Comparisons between the three groups were performed using one-way analysis of variance, and multiple comparisons between groups were performed using the Student-Newman-Keuls test. The Mantel-Cox log-rank test was used to assess survival. Significance was defined as *P* < 0.05.

## RESULTS

### Neonatal BALB/c mice were infected with EV-A71

**Fig 1:**
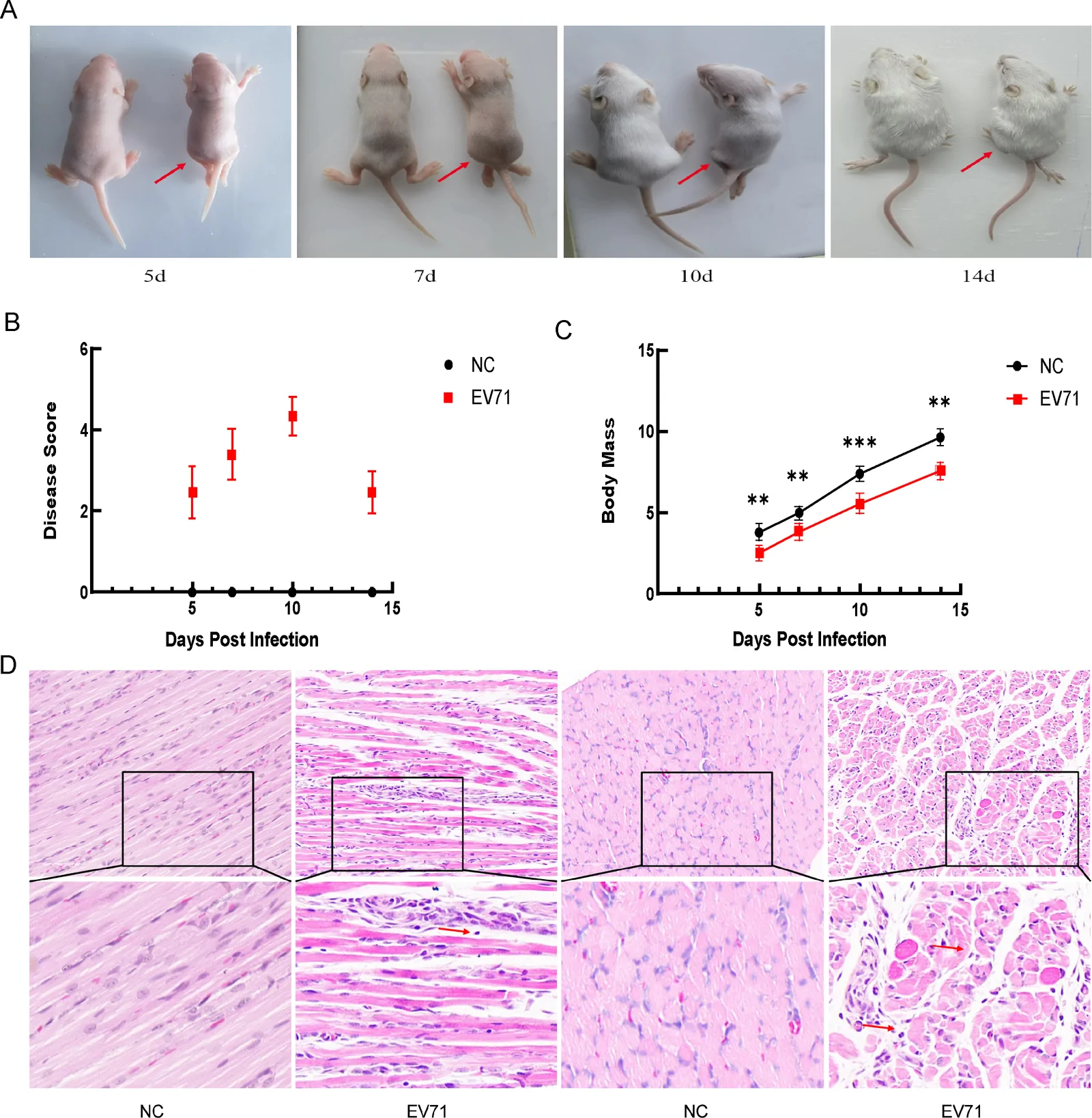
Infection of suckling BALB/c mice with EV-A71. (**A**) Normal BALB/c suckling mice on the left and EV-A71-infected BALB/c suckling mice on the right (indicated by red arrows). (**B**) Disease score of BALB/c suckling mice: NC group on day 5, *n* = 15; NC group on day 7, *n* = 15; NC group on day 10, *n* = 15; NC group on day 14, *n* = 15; 5 days after EV-A71 infection, *n* = 15; on day 7 after EV-A71 infection, *n* = 15; 10 days after EV-A71 infection, *n* = 15; on day 14 after EV-A71 infection, *n* = 15. (**C**) Body weight score of BALB/c suckling mice: NC: *n* = 15; EV-A71: *n* = 15; compared with the control group, the difference was statistically significant (**P < 0.01, ***P < 0.001). (**D**) Panel shows the pathology of skeletal muscle of neonatal BALB/c mice in sections along muscle fibers and in cross section. Samples were collected on 10 days after inoculation. (400×, 50 μm).

Compared with the normal control group, EV-A71-infected suckling mice showed thin stature, weight loss, hind limb weakness, paralysis, and sparse hair (Fig. 1A). As the time post-EV-A71 infection increased, the weight of the suckling mice in the infected group increased, while the clinical symptom scores gradually decreased (Fig. 1B and C). There were significant differences between the normal group and the EV-A71-infected group. HE staining results of skeletal muscle tissue of EV-A71-infected mice showed disordered muscle fiber arrangement and a large number of inflammatory cell infiltrations, interstitial edema, muscle bundle rupture and dissolution, and reduced or disappeared striated muscle tissue. The skeletal muscle tissue structure of the suckling mice in the normal group was intact, without inflammatory cell infiltration (Fig. 1D). These results show that EV-A71-infected BALB/c suckling mice have obvious appearance and pathological changes.

### EV-A71 infection caused skeletal muscle injury in neonatal BALB/c mice

**Fig 2:**
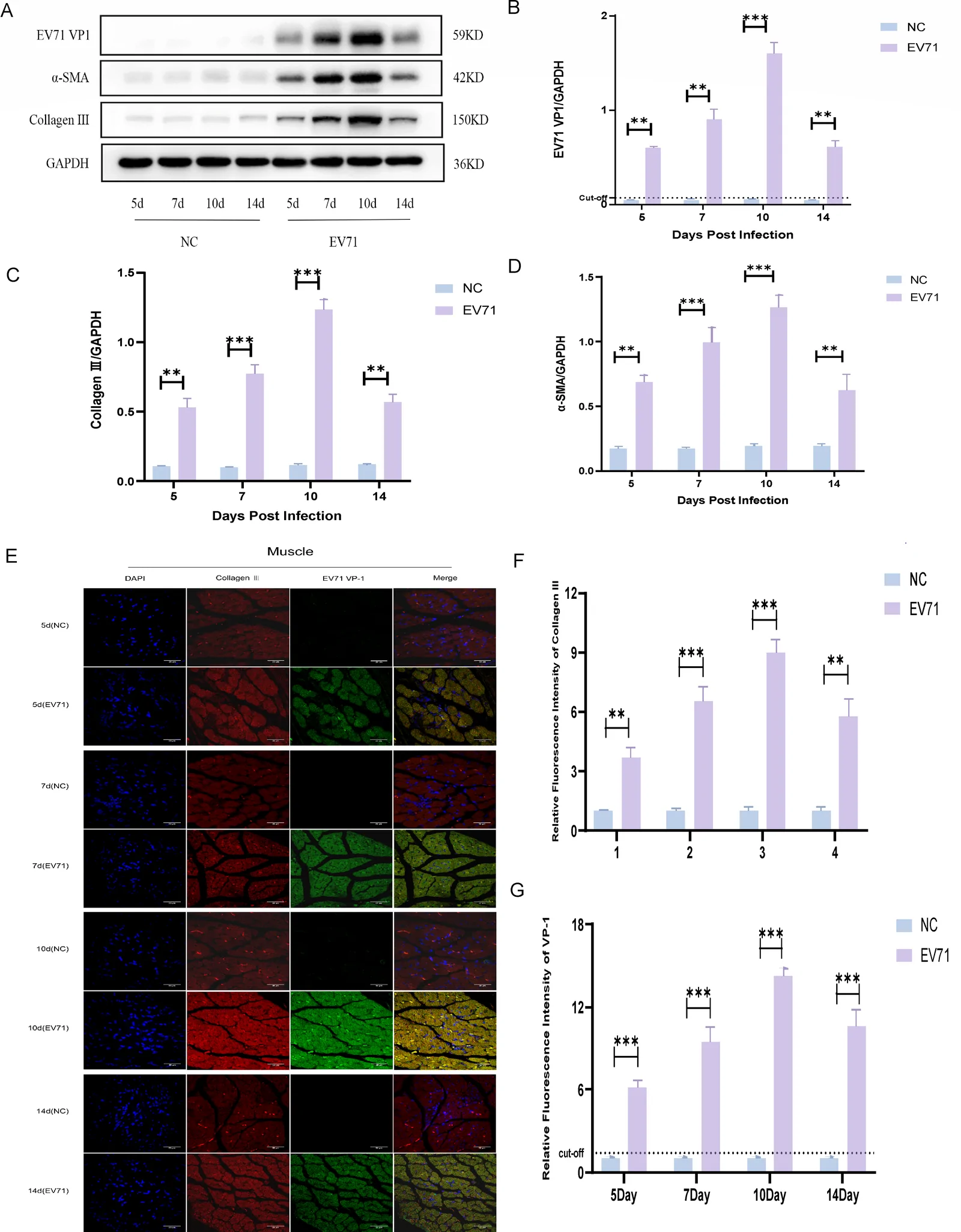
Effect of EV-A71 infection on skeletal muscle injury in neonatal BALB/c mice. (**A**) EV-A71 VP1, α-SMA, and type III collagen protein levels in skeletal muscle tissues from normal and EV-A71-infected BALB/c suckling mice at different days post-infection were analyzed by Western blotting. (**B**) The ratio of EV-A71 VP1/GAPDH in item **A** was analyzed by statistical analysis. The horizontal dotted line indicates the cut-off value for EV-A71 VP1 detection in panel B, which was 0.0665. (**C**) The ratio of type III collagen/GAPDH in item **A** was analyzed by statistical analysis. (**D**) The ratio of α-SMA/GAPDH in item **A** was analyzed by statistical analysis. (**E**) EV-A71 VP1 and type III collagen protein levels in skeletal muscle tissues from normal and EV-A71-infected BALB/c suckling mice at different days post-infection were measured by immunofluorescence staining. (**F**) The relative fluorescence intensity of type III collagen in item **E** was analyzed by statistical analysis. (**G**) The relative fluorescence intensity of EV-A71 VP1 in item **E** was analyzed by statistical analysis. The horizontal dotted line indicates the cut-off value for EV-A71 VP1 detection in panel G, which was 1.4166. The cut-off values were calculated as the mean value of the NC group plus three standard deviations. Values below these thresholds were considered background-level signals. The difference was statistically significant compared with the control group (**P < 0.01, ***P < 0.001).

VP1, as the signature protein of the EV-A71 virus strain, is not expressed in the skeletal muscle tissue of BALB/c neonatal mice in the normal group. However, after EV-A71 infection, VP1 protein levels increased significantly and reached a peak on day 10 of infection (Fig. 2A and B). To further explore whether EV-A71 infection can cause skeletal muscle damage, we detected the levels of skeletal muscle damage-related proteins on days 5, 7, 10, and 14 after infection. Western blot results showed that EV-A71 infection caused the expression of collagen III and α-SMA in the skeletal muscle tissue of suckling mice to be significantly higher than that in the normal control group (Fig. 2A through D). At the same time, immunofluorescence results also showed that the expression of collagen III and α-SMA was significantly increased in the skeletal muscle tissue of EV-A71-infected neonatal mice (Fig. 2E through G). These results indicate that EV-A71 infection leads to skeletal muscle damage.

### EV-A71 infection upregulates the TLR2/NF-κB signaling pathway

**Fig 3:**
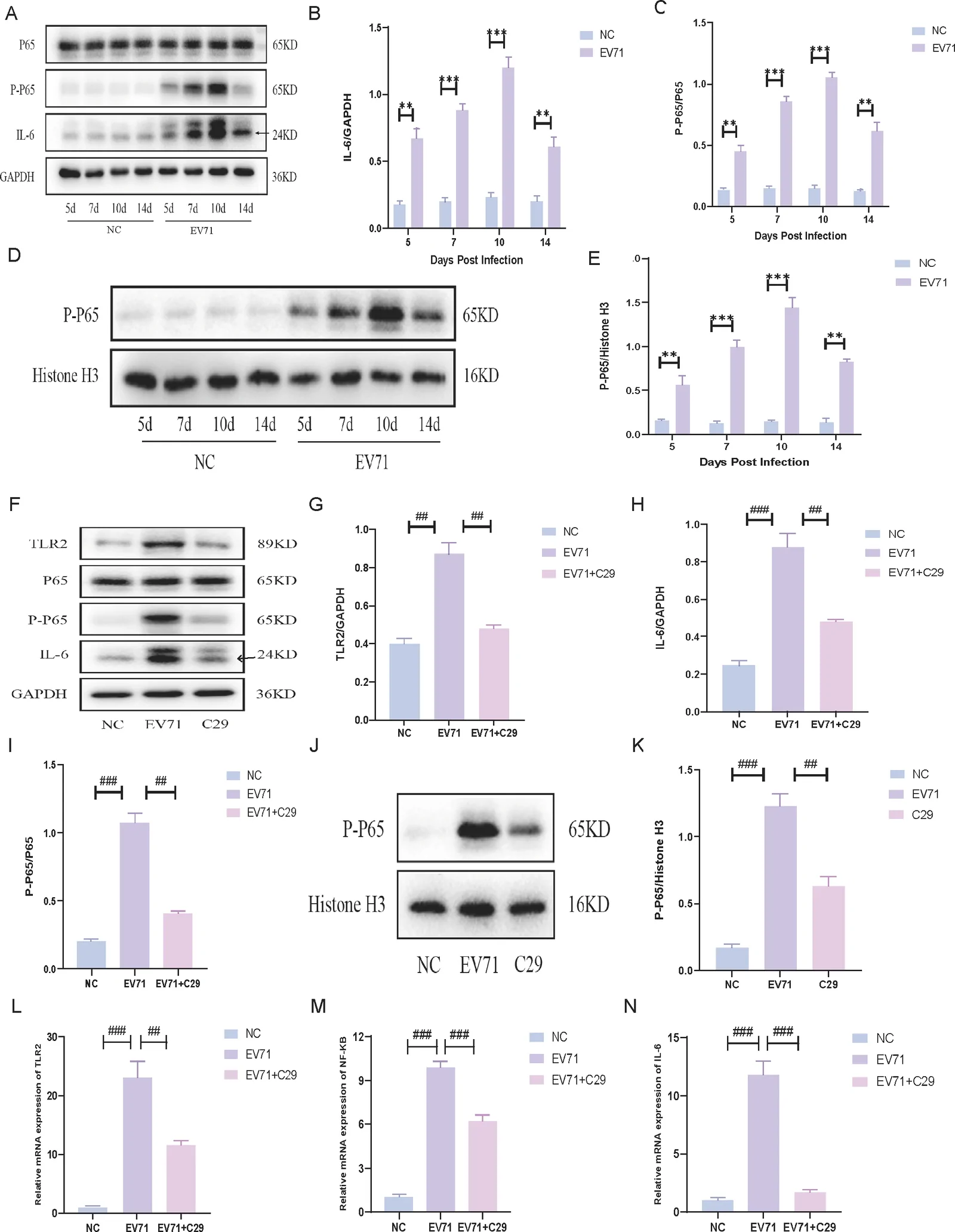
Effect of EV-A71 on TLR2/NF-κB signaling. (**A**) Western blotting analysis of NF-κB (P-P65), NF-κB (P65), and IL-6 proteins in skeletal muscle of neonatal BALB/c mice. (**B**) Results of statistical analysis of IL-6/GAPDH in item **A**. The band at approximately 24 kDa, corresponding to the predicted molecular weight of IL-6, was selected for densitometric analysis. (**C**) Results of statistical analysis of P-P65/P65 in item **A**. (**D**) Western blotting analysis of NF-κB (P-P65) in skeletal muscle of neonatal BALB/c mice after nuclear protein extraction with a nuclear protein extraction kit. (**E**) Results of statistical analysis of P-P65/Histone H3 in item **D**. (**F**) Western blotting analysis of TLR2, NF-κB (P-P65), NF-κB (P65), and IL-6 proteins in skeletal muscle of 10-day-old BALB/c suckling mice. (**G**) Results of statistical analysis of TLR2/GAPDH in item (**F**). (**H**) Results of statistical analysis of IL-6/GAPDH in item **F**. The band at approximately 24 kDa, corresponding to the predicted molecular weight of IL-6, was selected for densitometric analysis. (**I**) Results of statistical analysis of P-P65/P65 in item **F**. (**J**) Western blotting analysis of NF-κB (P-P65) in skeletal muscle of 10-day-old BALB/c suckling mice after nuclear protein extraction with a nuclear protein extraction kit. (**K**) Results of statistical analysis of P-P65/Histone H3 in item **J**. (**L**) The expression of TLR2 mRNA in skeletal muscle of 10-day-old BALB/c suckling mice was measured by qRT-PCR. (**M**) NF-κB mRNA levels in skeletal muscle of 10-day-old BALB/c suckling mice were determined by qRT-PCR. (**N**) IL-6 mRNA levels in skeletal muscle of 10-day-old BALB/c suckling mice were measured by qRT-PCR. The differences were statistically significant (compared with the control group, **P < 0.01, ***P < 0.001; compared with the EV-A71 infection group, ##*P* < 0.01, ###*P* < 0.001).

The study found that after EV-A71 infection, the expression levels of NF-κB (P-P65) and IL-6 in the skeletal muscle tissue of suckling mice increased significantly, reaching the highest value on the 10th day (Fig. 3A through C). After nuclear protein was extracted using a nuclear protein extraction kit, a significant increase in nuclear NF-κB (P-P65) protein levels was detected in the skeletal muscle tissue of EV-A71-infected BALB/c neonatal mice (Fig. 3D and E). Immunofluorescence staining and Western blot results showed that after intervention with the TLR2 inhibitor C29, the protein expression levels of TLR2, NF-κB (P-P65), and IL-6 in the skeletal muscle tissue of EV-A71-infected 10-day-old BALB/c suckling mice were significantly reduced (Fig. 3F through K and 4). qRT-PCR results showed that compared with EV-A71-infected suckling mouse tissues, the mRNA levels in the TLR2 inhibitor group were also significantly reduced (Fig. 3L through N). Taken together, these results suggest that the TLR2/NF-κB signaling pathway is involved in EV-A71 infection-induced skeletal muscle inflammation and injury.

**Fig 4:**
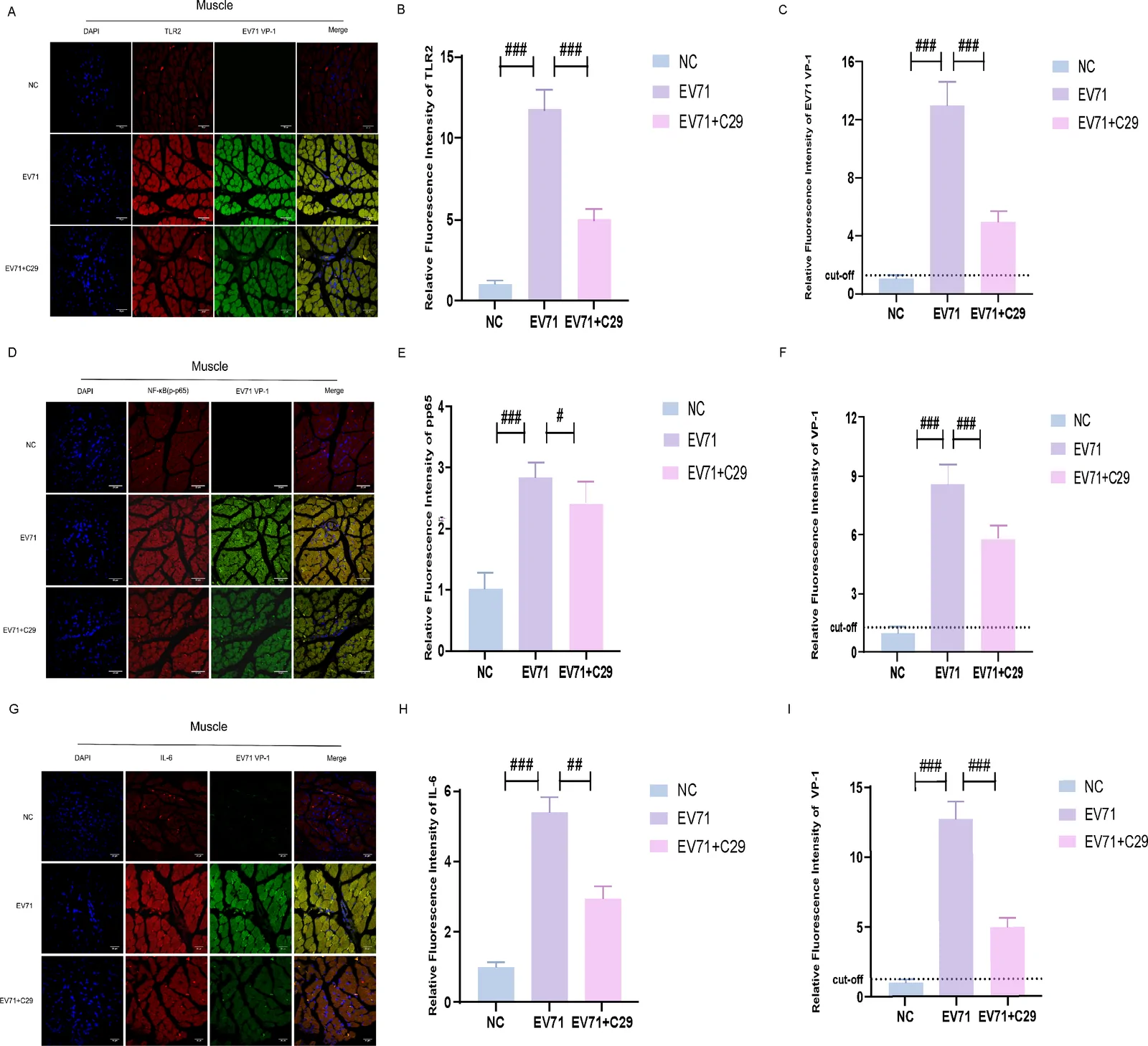
Impact of EV-A71 on TLR2/NF-κB signaling. (**A**) TLR2 and EV-A71 VP1 levels measured by IF staining in skeletal muscle of 10-day-old BALB/c suckling mice. (**B**) The relative fluorescence intensity of TLR2 in item (**A**) was analyzed by statistical analysis. (**C**) The relative fluorescence intensity of EV-A71 VP1 in item **A** was analyzed by statistical analysis. The horizontal dotted line indicates the cut-off value for EV-A71 VP1 detection in panel A, which was 1.2700. (**D**) IF staining was used to determine the levels of NF-κB (P-P65) and EV-A71 VP1 in skeletal muscle of 10-day-old BALB/c suckling mice. (**E**) The relative fluorescence intensity of NF-κB (P-P65) in item **D** was analyzed by statistical analysis. (**F**) The relative fluorescence intensity of EV-A71 VP1 in item **D** was analyzed by statistical analysis. The horizontal dotted line indicates the cut-off value for EV-A71 VP1 detection in panel D, which was 1.2760. (**G**) IF staining was used to measure the levels of IL-6 and EV-A71 VP1 in skeletal muscle of 10-day-old BALB/c suckling mice. (**H**) The relative fluorescence intensity of IL-6 in item **G** was analyzed by statistical analysis. (**I**) The relative fluorescence intensity of EV-A71 VP1 in item **G** was analyzed by statistical analysis. The horizontal dotted line indicates the cut-off value for EV-A71 VP1 detection in panel G, which was 1.2670. The cut-off values were calculated as the mean value of the NC group plus three standard deviations. Values below these thresholds were considered background-level signals. The difference was statistically significant (compared with EV-A71 infection group, #*P* < 0.05, ##*P* < 0.01, ###*P* < 0.001).

### The TLR2/NF-κB pathway is involved in EV-A71 infection-induced skeletal muscle inflammation and injury

**Fig 5:**
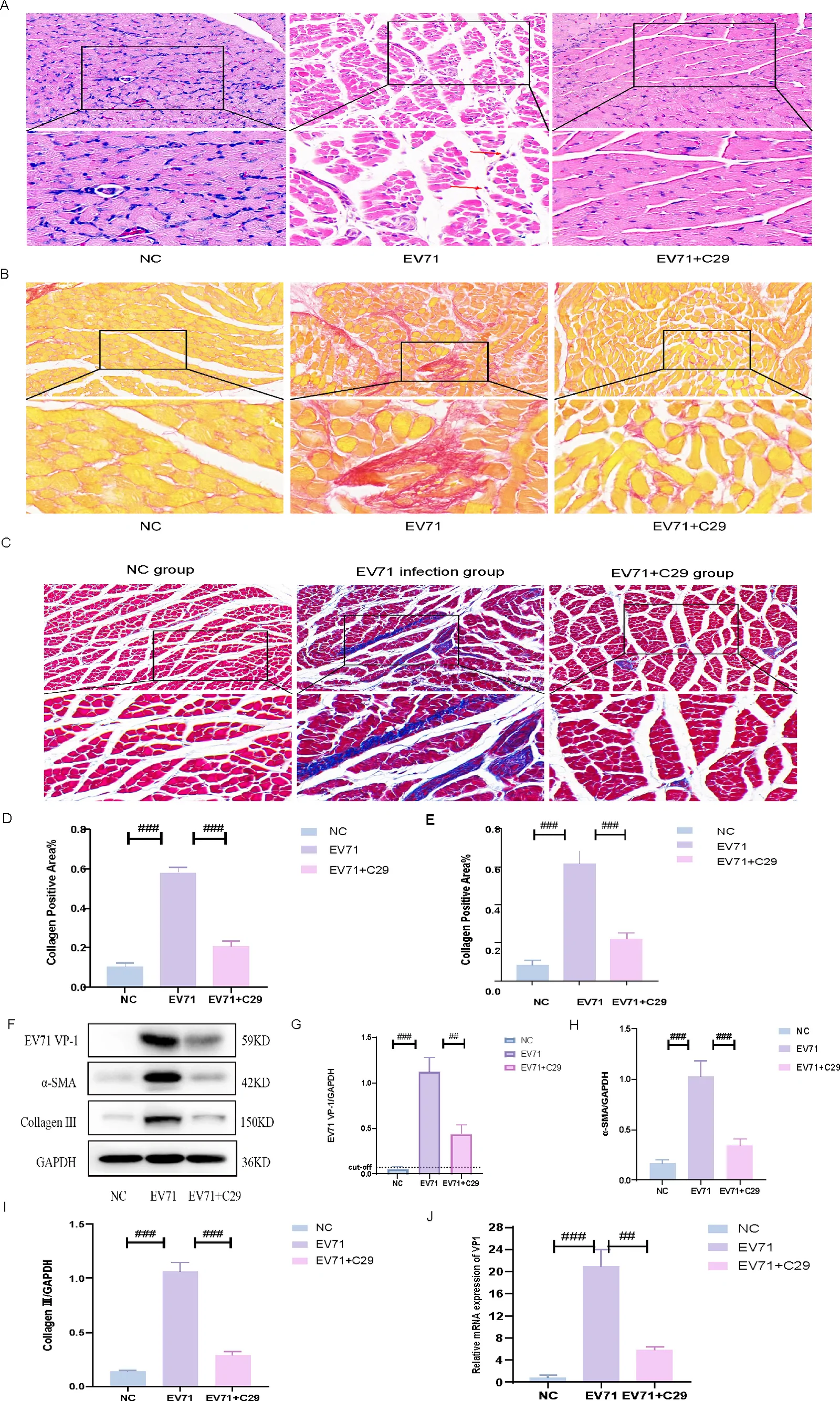
Effect of inhibition of TLR2/NF-κB signaling on pathological changes in skeletal muscle. (**A**) HE staining of skeletal muscle tissue from 10-day-old BALB/c suckling mice. (**B**) Sirius red staining of skeletal muscle tissue from 10-day-old BALB/c suckling mice. (**C**) Masson staining of skeletal muscle tissue from 10-day-old BALB/c suckling mice. (**D**) Analysis of the degree of fibrosis in item (**B**). (**E**) Analysis of the extent of fibrosis in item (**C**). (**F**) EV-A71 VP1, α-SMA, and type III collagen in skeletal muscle tissue of 10-day-old BALB/c suckling mice were analyzed by Western blotting. (**G**) The ratio of EV-A71 VP1/GAPDH in item **F** was analyzed by statistical analysis. The horizontal dotted line indicates the cut-off value for EV-A71 VP1 detection in panel F, which was 0.0730. (**H**) The ratio of α-SMA/GAPDH in item **F** was analyzed by statistical analysis. (**I**) The ratio of type III collagen/GAPDH in item **F** was analyzed by statistical analysis. (**J**) EV-A71 VP mRNA levels in skeletal muscle of 10-day-old BALB/c suckling mice were measured by qRT-PCR. The cut-off values were calculated as the mean value of the NC group plus three standard deviations. Values below these thresholds were considered background-level signals. The difference was statistically significant (compared with EV-A71 infection group, ##*P* < 0.01, ###*P* < 0.001).

The study in this paper has confirmed that EV-A71 infection upregulates the TLR2/NF-κB signaling pathway. In order to explore the effect of blocking TLR2 on the pathological changes of skeletal muscle tissue of BALB/c neonates induced by EV-A71, we conducted relevant experiments. Pathological results showed that EV-A71 infection caused pathological changes such as inflammatory cell infiltration in the skeletal muscle tissue of 10-day-old BALB/c suckling mice (Fig. 5A). In picrosirius red staining, we found that compared with the control group, collagen was significantly accumulated in the skeletal muscle tissue of EV-A71-infected 10-day-old BALB/c suckling mice, and large red fiber stripes were visible, while inhibition of the TLR2/NF-κB signaling pathway could alleviate the pathological changes of skeletal muscle induced by EV-A71 (Fig. 5B). In Masson trichrome staining, there was no collagen fiber deposition in the skeletal muscle tissue of 10-day-old BALB/c suckling mice in the normal group, and a large amount of blue collagen fibers were observed in the skeletal muscle tissue of EV-A71-infected 10-day-old BALB/c suckling mice. The blue collagen fiber stripes in the skeletal muscle tissue of 10-day-old BALB/c suckling mice in the TLR2 inhibition group were significantly reduced compared with the EV-A71-infected group (Fig. 5C). Previous studies have confirmed that collagen III and α-SMA play an important role in myocardial and lung injury. Other studies have also reported that TLR2 induces neurological symptoms during EV-A71 infection of 10-day-old BALB/c suckling mice. In this context, we further investigated whether skeletal muscle inflammation and injury caused by EV-A71 infection are mediated through the TLR2/NF-κB pathway. As shown in the figure, after intraperitoneal injection of the TLR2 inhibitor C29, we detected the changes in the protein and mRNA expression of VP1 and the protein expression of collagen III and α-SMA in the skeletal muscle tissue of EV-A71-infected 10-day-old BALB/c suckling mice. Immunofluorescence and Western blotting results showed that the protein levels of VP1, collagen III, and α-SMA were low in the normal group and significantly increased in the EV-A71 infection group. After the use of TLR2 inhibitors, the expression of VP1, collagen III, and α-SMA was significantly downregulated (Fig. 5F through I and 6). qRT-PCR results showed that the mRNA level of EV-A71 VP1 in the TLR2 inhibition group was significantly reduced compared with the EV-A71 infection group (Fig. 5J). Taken together, these results indicate that the TLR2/NF-κB signaling pathway plays a key role in skeletal muscle inflammation and injury caused by EV-A71 infection.

**Fig 6:**
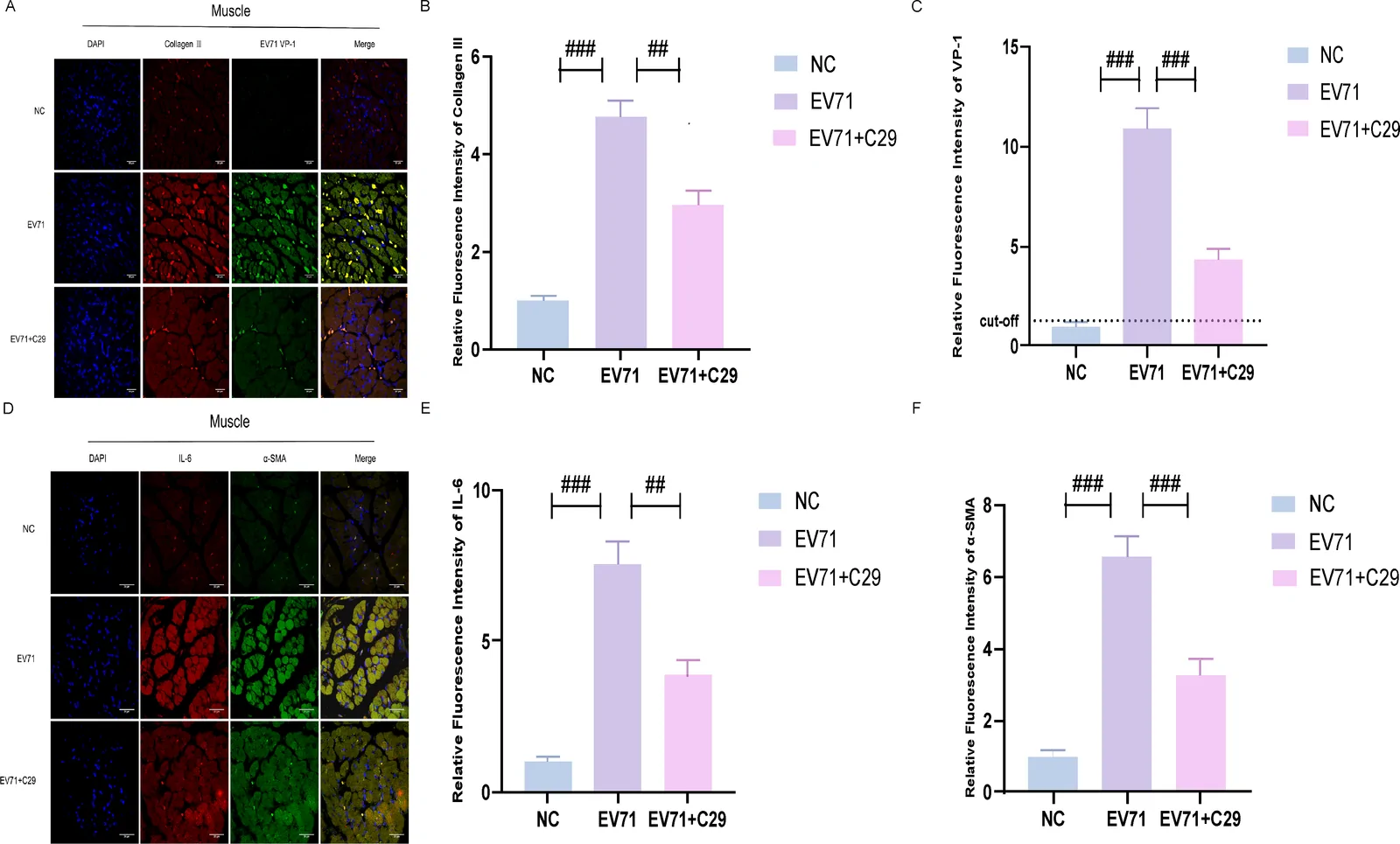
Impact of TLR2/NF-κB signaling inhibition on skeletal muscle pathology. (**A**) EV-A71 VP1 and type III collagen levels detected by IF staining in skeletal muscle of 10-day-old BALB/c suckling mice. (**B**) The relative fluorescence intensity of type III collagen in item **A** was analyzed by statistical analysis. (**C**) The relative fluorescence intensity of EV-A71 VP1 in item **A** was analyzed by statistical analysis. The horizontal dotted line indicates the cut-off value for EV-A71 VP1 detection in panel A, which was 1.2620. (**D**) IF staining was used to detect the levels of IL-6 and α-SMA in skeletal muscle of 10-day-old BALB/c suckling mice. (**E**) The relative fluorescence intensity of IL-6 in item **D** was analyzed by statistical analysis. (**F**) The relative fluorescence intensity of α-SMA in item **D** was analyzed by statistical analysis. The cut-off values were calculated as the mean value of the NC group plus three standard deviations. Values below these thresholds were considered background-level signals. The difference was statistically significant (compared with the EV-A71 infection group, ##*P* < 0.01, ###*P* < 0.001).

## DISCUSSION

EV-A71 is one of the major pathogens responsible for hand, foot, and mouth disease and can cause severe neurological and systemic complications in infants and young children (16, 17). In addition to central nervous system involvement, previous infection models in newborn animals have also suggested that skeletal muscle involvement is a significant pathological feature of EV-A71 infection; infected animals may exhibit changes such as limb weakness, paralysis, myositis, and structural damage to skeletal muscle (15, 18, 19). These findings suggest that skeletal muscle damage is not merely a secondary manifestation of systemic infection, but may be an important pathological component contributing to motor dysfunction and disease severity. In our study, we infected BALB/c suckling mice with an EV-A71 virus strain and found that, compared with the control group, EV-A71 infection induced pathological changes in the skeletal muscle of BALB/c suckling mice. From day 5 onward, suckling mice in the EV-A71-infected group exhibited significant clinical symptoms, including lethargy, emaciation, sparse fur, paralysis of both hind limbs, and sluggish movement. HE staining revealed that EV-A71 infection led to muscle fiber rupture, disorganized arrangement, and inflammatory cell infiltration in the skeletal muscle tissue of the suckling mice. EV-A71 infection resulted in elevated levels of inflammation-related proteins in skeletal muscle tissue: Western blot results showed that EV-A71 infection significantly upregulated the protein levels of EV-A71 VP1, NF-κB (P-P65), and IL-6 in skeletal muscle tissue, peaking on day 10 post-infection. These findings support the notion that skeletal muscle is a key target tissue in this infection model and suggest that the local inflammatory response is closely associated with EV-A71-related muscle pathological damage.

Viral skeletal muscle injury can be driven by the direct action of the virus and host-mediated immunopathological responses, either individually or in combination (20, 21). Although activation of the innate immune system is necessary for viral recognition and antiviral defense, excessive or persistent inflammatory signaling may disrupt tissue homeostasis and exacerbate muscle damage (22–24). TLR2 is a membrane-associated pattern recognition receptor that links pathogen recognition to downstream inflammatory signaling; it is particularly closely associated with the MyD88/NF-κB pathway (25–28). This study demonstrates that both TLR2 expression and NF-κB-related signaling are significantly enhanced in skeletal muscle following EV-A71 infection, while treatment with the TLR2 inhibitor C29 reduces TLR2 and viral VP1 levels, suggesting that TLR2-related pathways are involved in the local inflammatory response following EV-A71 infection and may influence viral load. Given that C29 primarily inhibits downstream TLR2 signal transduction rather than directly degrading TLR2, the reduction in TLR2 expression following treatment with C29 suggests that downstream inflammatory signaling may be involved in maintaining or enhancing TLR2 expression. It is therefore hypothesized that, following the activation of TLR2 and NF-κB signaling by EV-A71, the pro-inflammatory cytokines and local inflammatory mediators produced may further increase TLR2 expression or responsiveness, thereby forming a positive-feedback mechanism of inflammatory amplification that promotes persistent muscle inflammation and tissue damage.

A notable finding in this study was that C29 also reduced EV-A71 viral signaling. RT-qPCR results showed that C29 reduced EV-A71 VP1 RNA levels in skeletal muscle tissue infected with EV-A71. These results suggest that C29 reduces signaling associated with EV-A71 viral replication. Previous *in vitro* studies have shown that TLR2 and TLR2-containing heterodimers can activate the innate antiviral immune response and inhibit EV-A71 replication in specific cellular models (29). Therefore, the decrease in VP1 levels following C29 treatment in this study should not be simply interpreted as TLR2 directly promoting EV-A71 replication; rather, it is more likely to reflect a context-dependent balance between antiviral defenses, inflammatory damage, and the local cellular environment.

One possible explanation is that TLR2-mediated signaling plays a stage-dependent role during EV-A71 infection. On the one hand, TLR2 activation may contribute to the innate antiviral immune response (29). On the other hand, previous studies have shown that TLR2-related signaling can regulate glycolysis in immune cells via context-dependent pathways such as Akt–mTOR, accompanied by alterations in mitochondrial respiration, ATP production, and biosynthetic status (30, 31). Separate studies on EV-A71 have shown that viral infection can significantly remodeling the host cell’s glucose, glutamine/glutamate, and lipid metabolism, and that inhibition of PFKFB3-mediated glycolysis, glutamine metabolism, or fatty acid β-oxidation can all reduce viral replication (31–34). Furthermore, as a positive-strand RNA virus, EV-A71 relies on membrane-bound replicative organelles formed through the remodeling of the host endomembrane system for its RNA replication, and requires the involvement of multiple host proteins in the formation of these organelles, the assembly of the replicative complex, and viral RNA synthesis (35–38). Consequently, the decrease in VP1 levels following C29 treatment in this study may partly reflect changes in host metabolism and the inflammatory microenvironment resulting from the suppression of TLR2 signaling, thereby reducing the cellular conditions that support viral replication or persistence. At the same time, C29’s inhibition of the TLR2–MyD88–NF-κB signaling pathway may alleviate excessive inflammation and tissue damage. Consequently, the reduction in VP1 levels induced by C29 is more likely to represent a combined effect of inflammatory regulation, changes in host metabolism, and restricted viral replication, rather than being attributable to a single TLR2-dependent pro-viral metabolic mechanism.

During viral infection, the functions of different branches of the innate immune signaling pathways are not entirely identical. The pathway centered on interferon is typically associated with the establishment of an antiviral state, whereas NF-κB-mediated inflammatory signaling, when overactivated or persistently activated, may be more involved in the amplification of inflammation and tissue damage (39–41). In this study, conducted against the backdrop of EV-A71 infection in the skeletal muscle of newborn mice, no antiviral effect of TLR2-related signaling was observed. Following EV-A71 infection, TLR2 expression and NF-κB-related signaling increased in skeletal muscle; however, these markers decreased following C29 intervention, suggesting that the TLR2/NF-κB axis may primarily contribute to the maintenance and amplification of local inflammatory responses in this model. The decrease in VP1 levels following C29 treatment may reflect comprehensive changes in the local microenvironment—which influences the inflammatory state, the extent of cellular damage, and viral replication—following the suppression of TLR2 signaling. This further suggests that the net effect of TLR2 signaling may be dependent on cell type, tissue environment, and the stage of infection.

In addition to acute inflammatory damage, EV-A71 infection also promotes changes associated with skeletal muscle fibrosis. Persistent inflammation is a key driver of tissue remodeling, extracellular matrix deposition, and fibrosis (11, 42, 43). In this study, EV-A71 infection increased markers associated with collagen deposition and fibrosis, while treatment with C29 attenuated these changes. These results suggest that TLR2/NF-κB-mediated inflammation may link acute viral muscle injury to subsequent fibrotic remodeling. By inhibiting excessive TLR2-mediated inflammatory signaling, C29 may not only alleviate acute inflammatory damage but also limit abnormal skeletal muscle repair and the progression of fibrosis.

In summary, this study supports a model in which EV-A71 infection activates the TLR2/NF-κB signaling pathway in the skeletal muscle of suckling mice, thereby leading to amplified inflammation, muscle fiber damage, and fibrotic remodeling. By interrupting this pathological inflammatory cycle, C29 alleviates muscle inflammation, ameliorates tissue damage, reduces fibrotic changes, and decreases signaling associated with EV-A71 viral replication. Furthermore, studies have shown that EV-A71 infection may also influence cellular inflammatory responses and immune regulation through other pathways, such as the MAPK signaling pathway (29) and the JAK/STAT signaling pathway (44). The MAPK signaling pathway plays a crucial role in cellular stress responses and inflammatory reactions, while the JAK/STAT signaling pathway is a key route for the signal transduction of various cytokines and growth factors. EV-A71 infection may induce complex pathological changes through the cross-regulation of multiple signaling pathways, which represents one of the key areas for future research.

Although this study suggests, based on animal models, histopathology, and molecular analyses, that the TLR2/NF-κB axis is involved in EV-A71 infection-associated inflammatory damage and fibrotic remodeling of skeletal muscle, certain limitations remain. Firstly, this study was primarily based on a BALB/c suckling mouse infection model; as the immune development and skeletal muscle maturity of these mice differ from those of human pediatric patients, the relevant conclusions still require validation using clinical samples and a wider range of animal models. Secondly, while this study primarily employed the pharmacological inhibitor C29 to suppress TLR2 signaling, further experiments involving TLR2 gene knockout, cell-specific ablation, or *in vitro* genetic interventions are required to clarify the causal relationship. Furthermore, the specific mechanism underlying the reduction in VP1 levels following C29 treatment requires further elucidation; future studies could combine viral titer assays, analysis of replication intermediates, and time-course observations to distinguish its effects on viral replication, viral clearance, and the local inflammatory microenvironment. Subsequent research should also further elucidate the primary cellular sources of TLR2 signaling and incorporate assessments of long-term motor function, muscle fiber regeneration, and fibrosis outcomes, in order to provide a more comprehensive understanding of the mechanisms underlying EV-A71-associated skeletal muscle injury and the potential value of the TLR2/NF-κB pathway as a host-targeted intervention strategy.

The clinical significance of this study lies in its provision of new insights into the inflammatory basis of viral skeletal myopathies. EV-A71-associated muscle damage may not be explained solely by viral replication itself, but may instead result from the interaction between viral infection and a dysregulated host inflammatory response. By highlighting that the TLR2/NF-κB signaling pathway is a key contributor to EV-A71-associated skeletal muscle inflammation and remodeling, this study offers a perspective for host-targeted interventions. Modulating the overactivated TLR2-mediated inflammatory signaling may provide a potential strategy for alleviating virus-associated skeletal muscle damage and offer a theoretical basis for further understanding the pathogenesis of viral skeletal muscle disease.

### Conclusions

In conclusion, EV-A71 can induce local infiltration of pro-inflammatory factors, muscle fiber rupture, and upregulation of pro-inflammatory cytokines and extracellular matrix proteins in the skeletal muscle of lactating BALB/c mice. Inhibiting the TLR2/NF-κB signaling pathway exhibits a specific protective effect against EV-A71-induced skeletal muscle injury in lactating BALB/c mice. This inhibition reduces the secretion of pro-inflammatory factors, improves the inflammatory response in EV-A71-induced skeletal muscle, and consequently diminishes the secretion of fibrotic factors. Thus, EV-A71 may induce skeletal muscle injury in lactating BALB/c mice through the TLR2/NF-κB signaling pathway. Targeting this pathway may provide new insights into the shared molecular mechanisms underlying broad-spectrum virus-induced skeletal muscle injury and aid in the identification of potential therapeutic targets. Nonetheless, the mechanism of EV-A71-induced HFMD in children is intricate, and further research is warranted to investigate whether other pathways are involved in reducing skeletal muscle injury in EV-A71-infected mice.

## Data Availability

All relevant data supporting the findings of this study are available from the Figshare repository. The data can be accessed for review purposes via the following private link: https://doi.org/10.6084/m9.figshare.30019996.
